# New Method of Treating Yellow Fever

**Published:** 1888-09

**Authors:** Geo. M. Sternberg

**Affiliations:** Major and Surgeon United States Army; Johns Hopkins University, Baltimore, Md.


### PRELIMINARY NOTE UPON A NEW METHOD OF TREATING YELLOW FEVER.

BY GEO. M. STERNBERG,
Major and Surgeon United States Army.

In view of the recent announcenent that yellow fever has reappeared in Florida, and the possibility that it may soon prevail as an epidemic in that State, the writer has decided to report the favorable results recently attained in the treatment of the disease in Havana by a method proposed by him, although the number of cases is still comparatively small.

My recent researches in Havana have led me to think it very probable that in yellow fever, as in cholera, the specific micro-organism causing the disease is located in the alimentary canal. While this is not proved, it is demonstrated that, as a rule, no micro-organism capable of development in the culture-media usually employed by bacteriologists is present in the blood or tissues of those recently dead from yellow fever. This view naturally suggests intestinal antisepsis as a mode of treatment.

It is well known that in yellow fever the urine and the vomited matters are highly acid. I have also found the intestinal contents to have usually a more or less decided acid reaction. A microbe, therefore, capable of multiplying in the stomach and intestine in this disease must be able to grow in an acid medium. But aside from this theoretical reason for prescribing alkalies, the highly acid condition of the secretions furnishes an indication for such a treatment, and the writer has long desired an opportunity to see a thorough trial of a decidedly alkaline treatment.

These considerations induced me during a recent visit to Havana to propose the following formula :

R. Sodii bicarb. grammes x (gr. 150).
Hydrag. chlorid. corrosiv. centigrammes ii (3-10 gr).
Aquæ puræ, litre i (1 quart). M.
Sig.—50 grammes (about 1¾ oz.) every hour ; to be given *ice cold*.

This treatment was adopted by Dr. Raphael Wiess, house physician at the Garcini Hospital, and I have just received from him a record of twelve cases treated by the director of the hospital, Dr. Francis Cabera, and himself.

The complete temperature charts sent to me by Dr. Weiss sustain the diagnosis in all of these cases, and this is confirmed by Dr. Daniel M. Burgess, United States sanitary inspector at Havana, who writes me:

"HAVANA, July 18, 1888.

"MY DEAR DR. STERNBERG: I send you by this mail Dr. Weiss's report of twelve cases treated by the alkaline and bichloride method. Although they were all Spaniards, and were not directly under my care, I had sufficient supervision of the cases to satisfy me that they were all genuine yellow fever. It will be seen that they all recovered, and I will add that every case so far treated at the Garcini by that method has recovered. While these twelve cases were being treated, and a little before, eight cases were treated in the same institution by other methods, and five of the eight died."

Dr. Weiss writes me that in some of the more severe cases he increased the amount or bichloride to five centigrammes ($\frac{3}{4}$ gr.) per litre. He says:

" Administering to the sick the bichloride of mercury, according to your directions, the gastric phenomena are very much modified; the gastric sediments appear later, and in no case have they been entirely dark. In some cases, truly dangerous on account of the state of the patient, in a general sense, gastric sediments" (black vomit) " did not appear, nor even nausea. The pains in the stomach were not so severe."

In some of the cases Dr. Weiss added benzoate of sodium to the formula for its laxative effect, and administered daily two enæmas containing each a gramme (15 grs.) of the phenate of sodium.

Sherry wine and hypodermic injections of ether were given during the second stage, when the pulse was feeble and the condition of the patient seemed to call for a stimulant.

With reference to the urinary secretion, Dr. Weiss writes—

" The urine has always been abundant. In some cases the density lowered, but was compensated by a large quantity of urine. Albumen did not appear in some of the cases; in others the amount was small, and in others it reached two grammes to the litre. The albumen has always disappeared on the second or third day, and then the fever ceased."

Although Dr. Weiss has sent me the clinical records of only twelve cases, he says in his letter—

" I have dealt with thirteen sick from the beginning of the disease, and all have recovered. . . . Before using this new treatment, during the present season, we had eight cases which we treated from the first day by the old method" (mainly symptomatic), "and five of the number perished."

This mode of treatment will, of course, require a more extended trial before the results can be fairly compared with those obtained by other methods. I may remark, however, that in similar cases treated by the "expectant" method, the mortality rarely falls below 20 per cent., and in hospital practice is often 50 per cent., or even more.

Whether the favorable results are to be ascribed to the alkali, or to the antiseptic action of the bichloride, can only be determined by testing the two remedies separately; but I am very much disposed to ascribe the unusual absence of gastric disturbance, and the abundant secretion of urine noted, to the alkaline element of the treatment, and it is extremely doubtful whether the bichloride reaches the intestine in sufficient quantity to exercise any decided antiseptic effect in that portion of the alimentary canal. The bicarbonate should be free from carbonate of sodium, as the mercuric chloride is decomposed by this salt and by alkalies generally. It is possible that the biniodide might be substituted to advantage in the formula above given.

In some experiments made by the writer several years since to determine the comparative antisepetic value of the salts and oxides of mercury, the following results were obtained:

| | Active. | Failed. |
|---|---|---|
| Biniodide of mercury...... | 1 to 20,000 | 1 to 40,000 |
| Bichloride of mercury..... | 1 to 15,000 | 1 to 20,000 |
| Protiodide of mercury..... | 1 to 10,000 | 1 to 20,000 |
| Yellow oxide of mercury.... | 1 to 1,000 | 1 to 2,000 |
| Black oxide of mercury.... | 1 to 500 | 1 to 1,000 |
| Calomel.......................... | | 1 to 100 |
| Blue mass.......................... | | 1 to 100 |

"In every case the antiseptic was carefully weighed and added to one hundred cubic centimetres of beef-peptone solution, or of veal broth. A similar quantity of the culture-fluid was put up as a *temoin* without the addition of the antiseptic. As the oxides and iodides of mercury are insoluble in water, the bottle was repeatedly shaken in order to dissolve in the albuminous culture-fluid as much of the antiseptic as possible. An undissolved remnant could, however, be recognized at the bottom of the bottle after this repeated shaking. Two drops of broken-down beef-stock were added to each bottle to cause speedy putrefaction of the culture-fluid in the absence of a sufficiently potent inhibition of the developing power of the bacteria of putrefaction. In every case in the comparative experiment the culture-fluid became clouded, and had a putrefactive odor at the end of twenty-four hours.

"The first column in our table shows the proportion in which the culture-fluid was preserved from any appearance of decomposition for at least a week, the duration of the experiment. In the proportion given in the second column a decided inhibiting power was shown, except in the case of calomel and blue mass, which, in the proportion given (1 to 100), gave no evidence of antiseptic power. The other salts and oxides in the list prevented decomposition for twenty-four hours in the proportion given in the second column; and it was not until the second day that the bacteria of putrefaction commenced to form a cloud at the upper surface of the fluid, which gradually extended until the fluid had entirely broken down, usually by the third or fourth day. The bottles containing the biniodide (1 to 20,000) and the bichloride (1 to 15,000) have now been standing in the laboratory for three weeks, and are as transparent and free from odor as the day they were put up." *

The bichloride, the biniodide, and the oxides of mercury exhibited a decided inhibitory—antiseptic—effect upon the development of the bacteria of putrefaction in the proportion given in the second column, and we may use these figures as a guide in our efforts to secure intestinal antisepsis by means of these agents; although it is, of course, very uncertain how much of the soluble mercuric salt introduced into the stomach reaches the intestine in an active form—probably very little. In the formula which I gave to the physicians of the Garcini Hospital the amount of bichloride prescribed is really only in the proportion of 1 to 50,000 parts of the solution; but I considered it advisable to commence the treatment with an amount that was entirely safe, and to increase the dose afterwards if it was found to be prudent and desirable to do so. Dr. Weiss has, in fact, increased the dose in several severe cases to five centigrammes per litre—1 to 20,000. As all of these cases recovered, we may infer that when largely diluted, as in this formula, and given in small quantities at short intervals, it may be safely administered in these doses; but, as already intimated, I am rather disposed to attribute the favorable course of the disease to the full alkaline treatment than to any antiseptic effect of the bichloride in the intestine. In the stomach, however, I should expect a decided antiseptic effect from the medicine as administered; and perhaps this is, after all, what is most needed.

JOHNS HOPKINS UNIVERSITY, BALTIMORE, MD.

### WHAT KILLS THE CHILDREN.

Ignorance kills the children. Now and then accident lends a hand, as when some poor toddler tumbles into boiling water, or a weary, restless mother rolls

*From report of Committee on Disinfectants of the American Public Health Association, 1885.

